# Genomics and LC-MS Reveal Diverse Active Secondary Metabolites in *Bacillus amyloliquefaciens* WS-8

**DOI:** 10.4014/jmb.1906.06055

**Published:** 2019-10-10

**Authors:** Hongwei Liu, Yana Wang, Qingxia Yang, Wenya Zhao, Liting Cui, Buqing Wang, Liping Zhang, Huicai Cheng, Shuishan Song, Liping Zhang

**Affiliations:** 1 College of life science, Hebei University, Baoding 07002, P.R. China; 2 Institute of Biology, Hebei Academy of Science, Shijiazhuang 050081, P.R. China; 3 Main Crops Disease of Microbial Control Engineering Technology Research Center in Hebei Province, Shijiazhuang 050081, P.R. China; 4 Hebei Normal University, Shijiazhuang 05002, P.R. China

**Keywords:** *Bacillus amyloliquefaciens* WS-8, genome sequence, biosynthetic gene cluster, antifungal, plant growth promoting

## Abstract

*Bacillus amyloliquefaciens* is an important plant disease-preventing and growth-promoting microorganism. *B. amyloliquefaciens* WS-8 can stimulate plant growth and has strong antifungal properties. In this study, we sequenced the complete genome of *B. amyloliquefaciens* WS-8 by Pacific Biosciences RSII (PacBio) Single Molecule Real-Time (SMRT) sequencing. The genome consists of one chromosome (3,929,787 bp) and no additional plasmids. The main bacteriostatic substances were determined by genome, transcriptome, and mass spectrometry data. We thereby laid a theoretical foundation for the utilization of the strain. By genomic analysis, we identified 19 putative biosynthetic gene clusters for secondary metabolites, most of which are potentially involved in the biosynthesis of numerous bioactive metabolites, including difficidin, fengycin, and surfactin. Furthermore, a potential class II lanthipeptide biosynthetic gene cluster and genes that are involved in auxin biosynthesis were found. Through the analysis of transcriptome data, we found that the key bacteriostatic genes, as predicted in the genome, exhibited different levels of mRNA expression. Through metabolite isolation, purification, and exposure experiments, we found that a variety of metabolites of WS-8 exert an inhibitory effect on the necrotrophic fungus *Botrytis cinerea*, which causes gray mold; by mass spectrometry, we found that the main substances are mainly iturins and fengycins. Therefore, this strain has the potential to be utilized as an antifungal agent in agriculture.

## Introduction

Pathogenic microorganisms affecting plant health are a major and chronic threat to food production and ecosystem stability all over the world [1]. At present, chemical control is the main approach to control plant diseases [2]. However, the long-term use of chemical fungicides has resulted in serious and multiple resistance of pathogenic microorganisms. At the same time, pesticide residues have polluted the environment, endangered human health, and destroyed the ecological balance. Biological control has attracted more and more attention and played an increasingly important role in the world because of its advantages to the environment, ecology, and human health.

*Bacillus* spp. are an important source of plant disease-preventing and growth-promoting microorganisms. The prevention of plant disease is mainly achieved by secreting various antimicrobial substances [3]. Johnson *et al*. [4] discovered that *Bacillus subtilis* can produce antimicrobial substances, many of which, including polypeptides, lipopeptides and antimicrobial proteins, have been isolated from different *Bacillus* species. *Bacillus amyloliquefaciens*, a non-pathogenic bacterial species that is widespread in nature, is known to produce bioactive compounds and is capable to promote plant growth. It has therefore been used as a biological control agent in agriculture [1].

The whole genome of *B. amyloliquefaciens* FZB42 [5], published in 2007, has provided a deeper understanding of *B. amyloliquefaciens* and its capacity to produce multiple secondary metabolites, promoting its biological utilization. The major active substances of *B. amyloliquefaciens* are antifungal peptides. *B. amyloliquefaciens* can produce antifungal peptides by two main pathways, *i.e.*, the ribosome pathway and the non-ribosome pathway. As an ever-increasing number of genomes of different *B. amyloliquefaciens* strains are sequenced, marked differences have been found between gene clusters of secondary metabolites in different strains [6], and the active substances of some strains may be totally different.

**Fig. 1:**
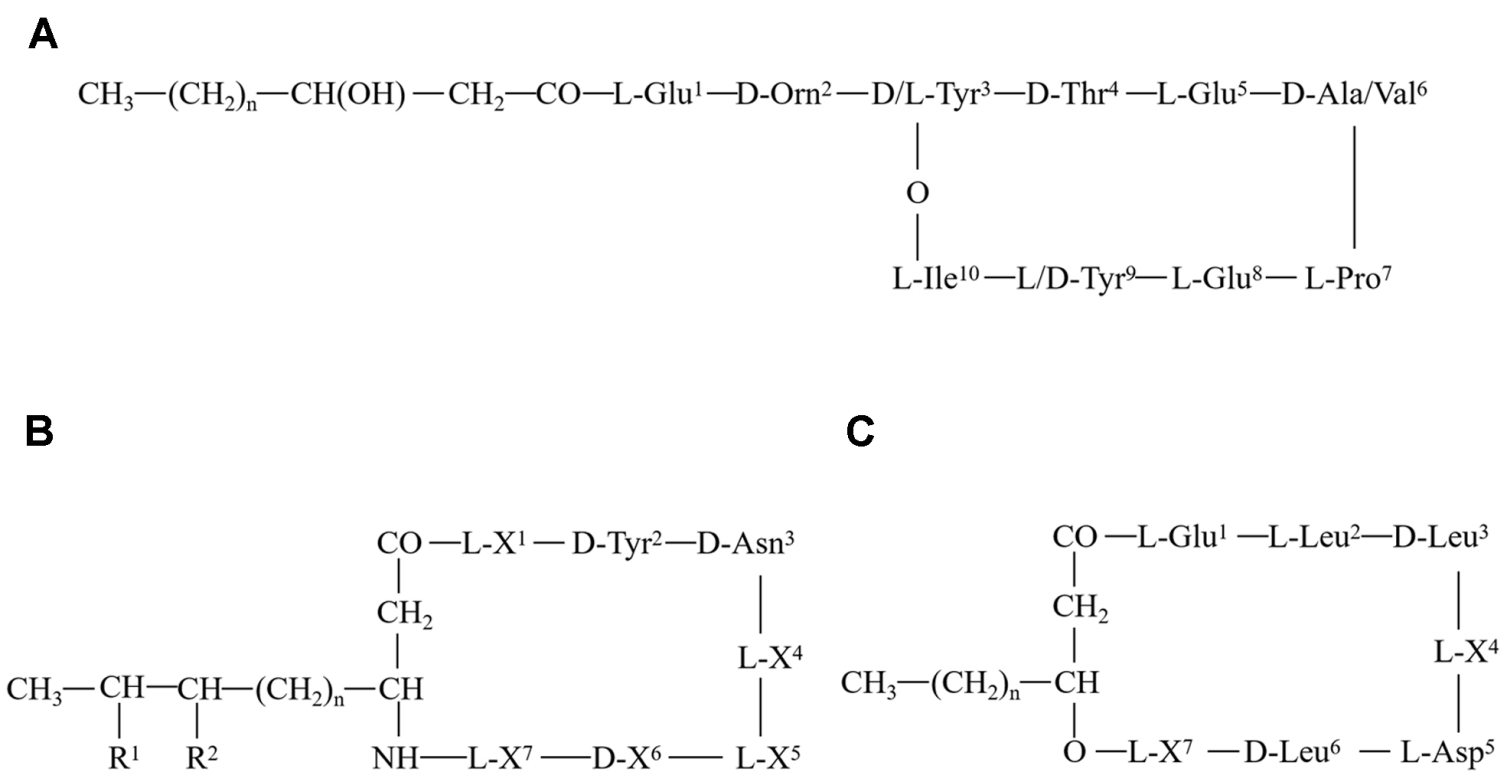
The chemical structure of fengycins (**A**), iturins (**B**), surfactins (**C**).

Fengycin, iturin, surfactin [7], difficidin, bacilysin [8], and macrolactin [9] are among the main antifungal peptides synthesized by the non-ribosomal pathway of *B. amyloliquefaciens*. The fengycins are a family of cyclic octapeptide-containing decapeptides (amino acid sequences are usually suggested as L-Glu-D-Orn-[D or L]-Tyr-D-Thr-L-Glu-D-[Ala or Val]-L-Pro-L-G1u-[L or D]-Tyr-L-Ile), which linked to a β-hydroxy fatty acid chain between C_12_ and C_19_ (Fig. 1A) [7]. Iturins and surfactins both are heptapeptides. The iturins are a family of cyclic heptapeptides (the 2nd and 3rd amino acids being always D-Tyr-D-Asn), which linked to a β-amino fatty acid chain between C_15_ and C_18_ (Fig. 1B) [10]. The surfactins are another family of cyclic heptapeptides (L-Glu-L-Leu-D-Leu-L-X4-L-Asp-D-Leu-L-X7, two of which are variable) [11], which linked to a β-amino fatty acid chain between C_13_ and C_17_ (Fig. 1C).

Although many strains have been used to control plant diseases, more efficient strains are still needed in agriculture. Before the use of a new strain as a biocontrol agent, it is recommendable to identify its main active substances. *B. amyloliquefaciens* WS-8, which can stimulate plant growth, has strong antifungal properties. Our preliminary study showed that the WS-8 strain exerts strong antagonistic activity against *Fulvia fulva* (Cooke) Cif and can promote growth of *Lycopersicon esculentum* Mill [12]. In order to determine the antifungal mechanism of *B. amyloliquefaciens* WS-8, we sequenced its complete genome. To elucidate the expression levels of core genes of secondary metabolic gene clusters, we also sequenced the transcriptome in the late logarithmic phase. Moreover, the anti-gray-mold compounds were isolated, purified, and characterized through chromatography and liquid chromatography-tandem mass spectrometry (LC-MS/MS). These results improve our understanding of the antifungal mechanisms of the WS-8 strain, and they will enable us to make better use of this strain as a fungicidal agent in agriculture.

## Materials and Methods

### Microorganisms and Culture Conditions

*B. amyloliquefaciens* WS-8 was isolated from hillside soil from Langya Mountain in Hebei, China, and stored at the China General Microbiology Culture Collection Centre (CGMCC: 11787). Luria Bertani (LB) broth medium (containing 10 g/l tryptone, 5 g/l yeast extract, and 5 g/l NaCl in distilled water) was used as growth medium. Bacteria were cultured at 32°C for 32 h with continuous shaking at 200 rpm.

### DNA Isolation, Genome Sequencing and Assembly

Genomic DNA of *B. amyloliquefaciens* WS-8 was extracted using the SDS method. A library with a 10-kb insert size was constructed for sequencing. The genome was sequenced using a PacBio RS II sequencing platform by Beijing Novogene Bioinformatics Technology Co., Ltd. The reads were assembled using the SMRT portal [13]. The complete genome sequence of *B. amyloliquefaciens* WS-8 has been submitted to GenBank under the accession number CP018200.

### Gene Prediction and Identification of Secondary Metabolite Clusters

Gene prediction was performed on the WS-8 genome assembly by GeneMarkS [14] with an integrated model which combines the GeneMarkS generated (native) and heuristic model parameters. Gene annotation was added by the NCBI Prokaryotic Genome Annotation Pipeline [15]. The genome comparison was carried out by RAST version 2.0 [16]. Genes potentially involved in the biosynthesis of antibiotics and secondary metabolites were identified using antiSMASH3.0 [17]. Genome overview was created by CGView Server to show the annotation information of WS-8 [18].

### Transcriptomic Analysis Using RNA-Seq

For RNA extraction, cells were cultivated for 32 h and harvested by centrifugation at 10,000 ×*g* for 5 min at 4°C. The pellets were immediately frozen in liquid nitrogen. Total RNA was extracted using an RNAprep Pure Cell/Bacteria Kit (TIANGEN BIOTECH, China) according to the manufacturer’s instructions. RNA integrity was assessed using the RNA Nano 6000 Assay Kit and a Bioanalyzer 2100 system (Agilent Technologies, USA). Sequencing libraries were generated using the NEBNext Ultra Directional RNA Library Prep Kit for Illumina (NEB, USA) following the manufacturer’s recommendations. The clustering of the index-coded samples was performed on a cBot Cluster Generation System using TruSeq PE Cluster Kit v3-cBot-HS (Illumia) according to the manufacturer’s instructions. After cluster generation, the library preparations were sequenced on an Illumina Hiseq 4000 platform and paired-end reads were generated.

Raw data (raw reads, fastq files) were first processed using in-house Perl scripts from Beijing Novogene Bioinformatics Technology Co., Ltd. In this step, clean data (clean reads) were obtained by removing reads containing adapters, reads containing poly-N tails, and low-quality reads from the raw data. Q20, Q30, and GC content of the clean data were calculated. All of the downstream analyses were based on the high-quality clean data. The remaining clear reads were mapped to the reference genome of WS-8 using Bowtie2 software based on the local alignment algorithm [19]. HTSeq v0.6.1 was used to count the reads mapped to each gene. The fragments per kilobase of transcripts per million mapped reads (FPKM) of each gene was calculated based on the length of the gene and read count mapped to this gene [20]. The transcriptomic data of *B. amyloliquefaciens* WS-8 has been submitted to GenBank under the accession number SRX6564092.

### Purification of Anti-Gray-Mold Compounds

The strain WS-8 was grown in nutrient broth (NB) at 32°C for 32 h. After centrifugation (10,000 rpm, 30 min, and 4°C), cells were removed and the cell-free supernatant (4 liters) was shaken with 200 g of Amberlite XAD-7HP (Sigma, USA) for 12 h at 18°C. The antimicrobial crude extract (CE) was obtained following the protocol of Xin [21]. Further purification was carried out by HPLC (SHIMADZU LC-20A, Japan) with a C_18_ column (250 × 4.6 mm, 5 µm; WONDASIL, Japan) at room temperature. The mobile phase consisted of acetonitrile and HPLC-grade water (with 0.1%trifluoroacetic acid [TFA]). A linear gradient was used for elution at a flow rate of 1 ml/min as follows: 0-60 min, from 10% to 80%acetonitrile (linear gradient); 60-65 min, from 80% to 90% acetonitrile (linear gradient); 65-75 min, 90% acetonitrile (isocratic); 75-80 min, from 90% to 10% acetonitrile (linear gradient); 80-90 min, 10%acetonitrile (isocratic). Elution was monitored by determining absorbance at 214 nm, and fractions were manually collected each minute. Using the agar well diffusion method [22, 23], fractions with anti-gray-mold (*Botrytis cinerea*) activity were detected and selected for LC-MS/MS analysis.

### LC-MS/MS Analysis

Liquid chromatograph-mass spectrometry (LC-MS) was performed by the Thermo Fisher UltiMate 3000 UPLC/Q-Exactive Orbitrap MS system. The UHPLC conditions were as follows: Thermo GOLD HYPERSIL column (C_18_, 50 × 2.1 mm, 1.9 µm; Thermo), eluent A was H_2_O/0.1% formic acid, eluent B was CH_3_OH/0.1% formic acid, flow rate was 300 µl/min, elution gradient was 70%A/30%B to 10%A/90%B, 20 min. The MS operating conditions were as follows: the temperature was 300°C, a sheath gas rate of 35 l/min, auxiliary gas rate of 35 l/min, electrospray voltage of 3.0 kV for positive full scan mode, and an *m/z* range of 100 - 3000. Detailed sequence information for antimicrobial peptides was obtained using the targeted MS/MS mode. The target ion was isolated and fragmented by adding a voltage of 35 V.

## Results

### General Genome Features of *B. amyloliquefaciens* WS-8

**Fig. 2:**
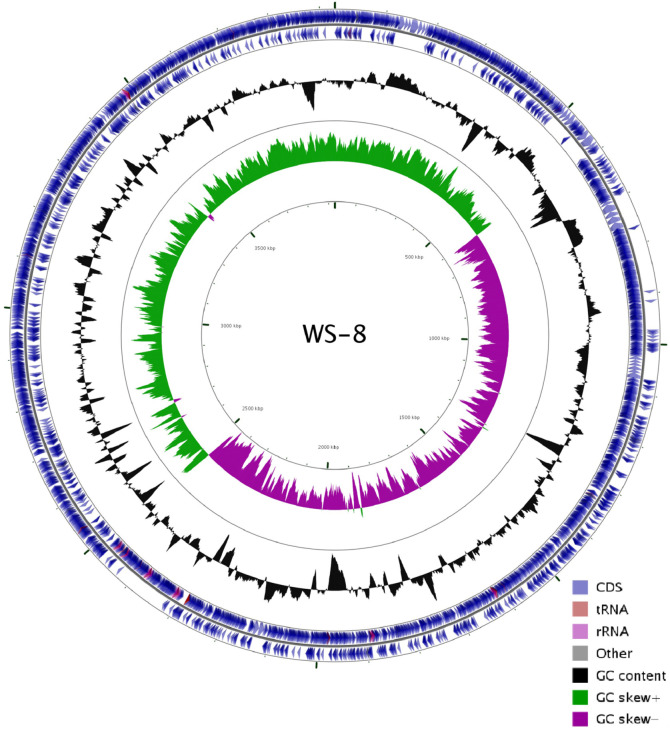
Circular genome map of *B. amyloliquefaciens* WS-8. The circular map consists of 5 circles. From the outermost circle inwards, circle (1) and circle (2) show the coding gene distributions in the forward strand and the backward strand, respectively, including tRNA (brown), rRNA (pink), and other (gray); and circle (3) shows GC content; circle (4) and circle (5) show GC skew+ and GC skew-, respectively.

A total of 90,776 reads, with approximately 311-fold coverage (approximately 1.227 G), were obtained. Compared to the type strain *B. amyloliquefaciens* FZB42, they constructed a library with fragments of 1.5 to 3.0 kbp, and abtained a total of 40,000 sequences [5]. Our sequencing coverage is at least 6 times more than the type stain FZB42. With the increase of sequencing coverage, the error of sequencing can be corrected, and the error rate of the whole genome can be reduced. The reads were assembled using the SMRT portal. The complete genome of WS-8 is composed of one gapless circular chromosome of 3,929,787 bp, almost the same as the genome of *B. amyloliquefaciens* FZB42 (3,918,589 bp) [5]. It shows a G+C content of 46.5%, and no plasmid was found. The genome contains 3895 predicted genes, 3777 protein coding genes, 107 pseudogenes, 86 tRNA genes, 27 rRNA genes, and 5 ncRNA genes (Table 1 and Fig. 2). Meanwhile, 32 long tandem repeats, 28 transposons, 32 long interspersed nuclear elements, 7 short interspersed nuclear elements, and 152 tandem repeat sequences were also identified in the genome. Among the identified genes, 2,895, 2,727, and 2,307 genes were classified to functional categories based on clusters of, orthologous genes of proteins (COG) [24], Kyoto Encyclopedia of Genes and Genomes designation (KEGG) [25], and gene ontology (GO) [26], respectively.

Using RAST version 2.0 under the Compare Metabolic Reconstruction model, a total of 2,428 functioning parts genes were found, 170 and 108 unique genes were identified from *B. amyloliquefaciens* WS-8 and *B. amyloliquefaciens* FZB42 respectively.

### Functional Gene Annotation

**Fig. 3:**
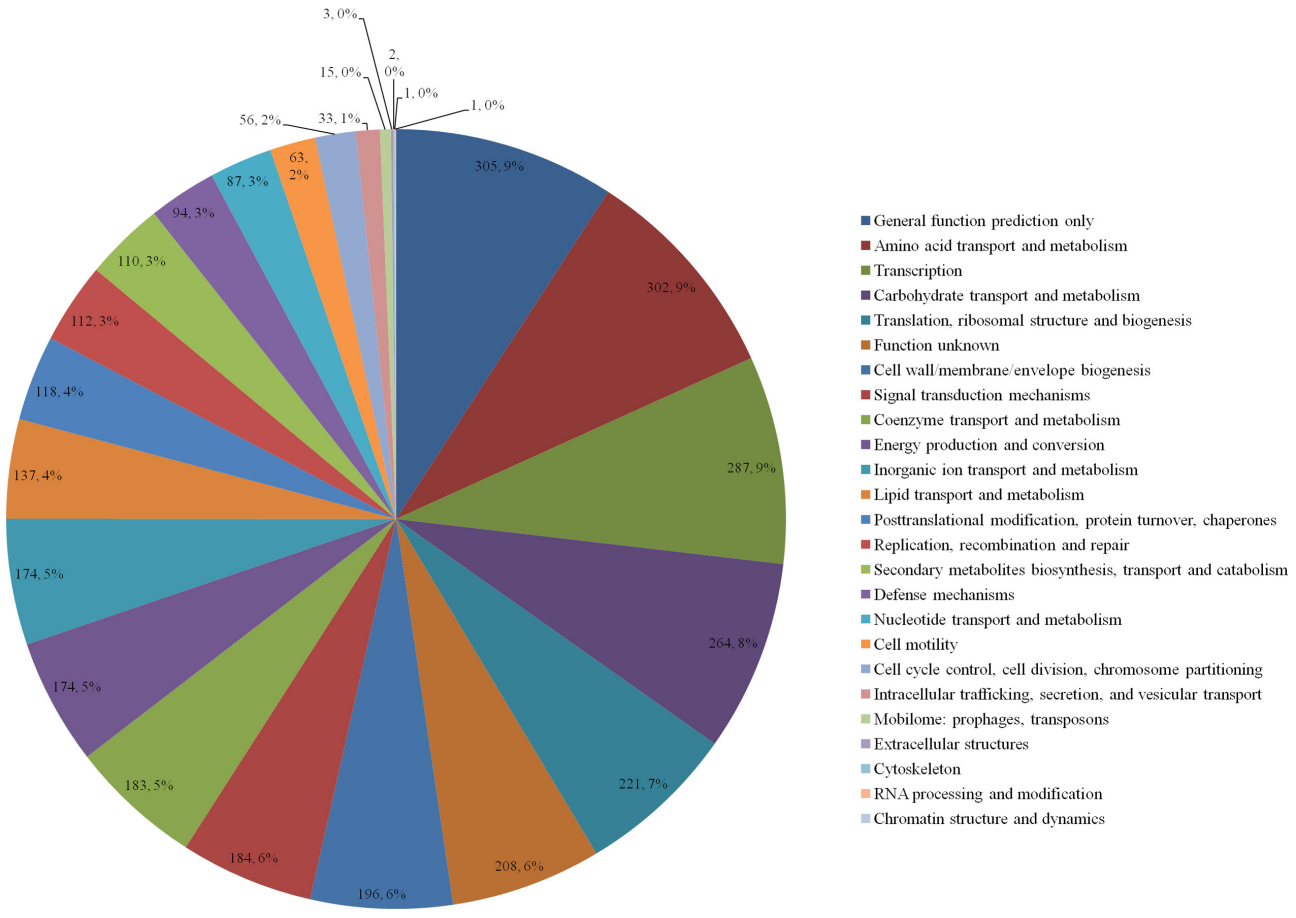
COG categories of *B. amyloliquefaciens* WS-8.

**Function and classification of COG.** The COG functional categories of the complete genome sequence of *B. amyloliquefaciens* WS-8 are shown in Fig. 3. Among the 3,895 genes, 2,895 genes were classified into COG categories. The major categories of *B. amyloliquefaciens* WS-8 were general function prediction only, amino acid transport and metabolism, transcription, carbohydrate transport and metabolism, translation, ribosomal structure and biogenesis, function unknown, and cell wall/membrane/envelope biogenesis. Additionally, the genome contains genes probably involved in the promotion of plant growth, *i.e.*, 31 genes for nitrogen metabolism, 40 genes for sulfur metabolism, 33 genes for phosphorus metabolism, 10 genes for potassium metabolism, and 4 genes for auxin biosynthesis.

**Identification of secondary metabolite clusters with antiSMASH.** After annotation, we identified genes and gene clusters related to the biosynthesis of interesting secondary metabolites using antiSMASH. We found 19 putative biosynthetic gene clusters for secondary metabolites (Table 2), *i.e.*, seven microcins, four non-ribosomal peptides, four polyketides, two terpenes, one lantipeptide, and one other metabolite. Seven putative gene clusters showed high similarity (> 70% of genes showing similarity) to reported difficidin, fengycin, bacillaene, macrolactin, surfactin, bacilysin, and bacillibactin biosynthesis gene clusters. Cluster 9 showed 7% similarity to butirosin, and the other gene clusters were not similar to any known cluster. Additionally, we identified a potential class II lanthipeptide biosynthetic pathway. In this gene cluster, we found three LanA-like (precursor peptide) genes, two LanM-like (modification enzyme) genes, one LanT-like (transporter, with a peptidase domain) gene, one LanI-like (immunity protein) gene, and five regulatory element genes [27, 28].

### Transcriptome Analysis of Secondary Metabolite Clusters

The transcriptome of stationary phase of the strain WS-8 was sequenced with the HiSeq4000 sequencing platform. A total of 9,006,772 clean Reads and 1.35 G clean data were obtained. The Q20 and Q30 of clean data were 98.07% and 94.69%, respectively. The GC content of the transcriptome was 48.08%. About 97.97% of the clean reads were mapped to the WS-8 genome. Using the annotation of antiSMASH for mapping and FPKM values to indicate the expression levels, the mapped genes were classified into four groups (Table 3). Using a threshold of FPKM > 1 to define potential gene expression [29], 3,540 expressed genes were sequenced. As shown in Table 3, more than 93% of the genes were expressed in the stationary phase. Most genes were expressed at a medium level, and more than 13% of the genes were expressed at a high level. In addition, there were 277 non-expressed genes.

**Fig. 4:**
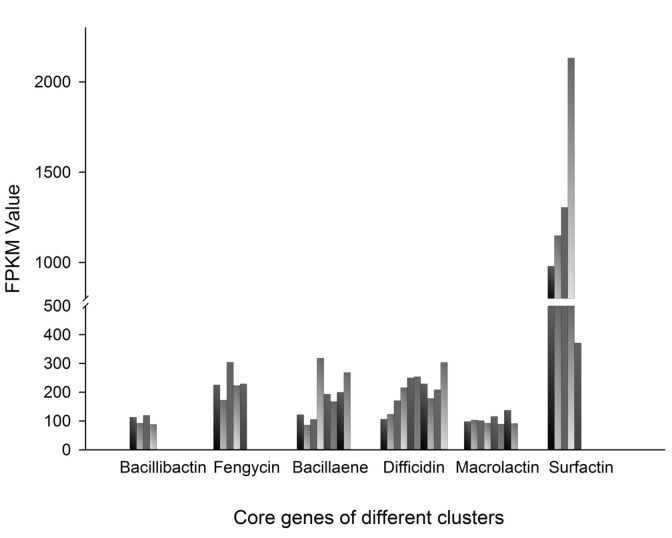
FPKM values of core genes of different gene clusters.

Using the FPKM value, the expression levels of core genes in gene clusters related to antibacterial substances were analyzed. We found that core genes of six gene clusters, which are homologous to bacillibactin, fengycin, bacillaene, difficidin, macrolactin, and surfactin biosynthetic gene cluster were all expressed (Fig. 4). All of the bacillibactin genes (bsuF, bsuB, bsuE, and bsuA), fengycin genes (fenA, fenB, fenC, fenD, and fenE), bacillaene genes (*baeE, baeD, baeC, baeR, baeN, baeM, baeL*, and *baeJ*), difficidin genes (*difA, difD, difE, difF, difG, difH, difI, difJ, difK*, and *difL*), and macrolactin genes (*mlnA, mlnB, mlnC, mlnD, mlnE, mlnF, mlnG*, and *mlnH*) were expressed at a medium level. In the surfactin gene cluster, there were four highly expressed genes (*srf'AA, srf'AB, srf'AC, srf'AD*), and one medium expressed gene (*sfp*). These data suggest that the products of these gene clusters may exist in WS-8. For the lantipeptide gene cluster, all of the precursor peptide genes, synthetase genes, and transport genes were also expressed.

### Purification and UPLC-MS Analysis of Antibacterial Substances from WS-8

**Fig. 5:**
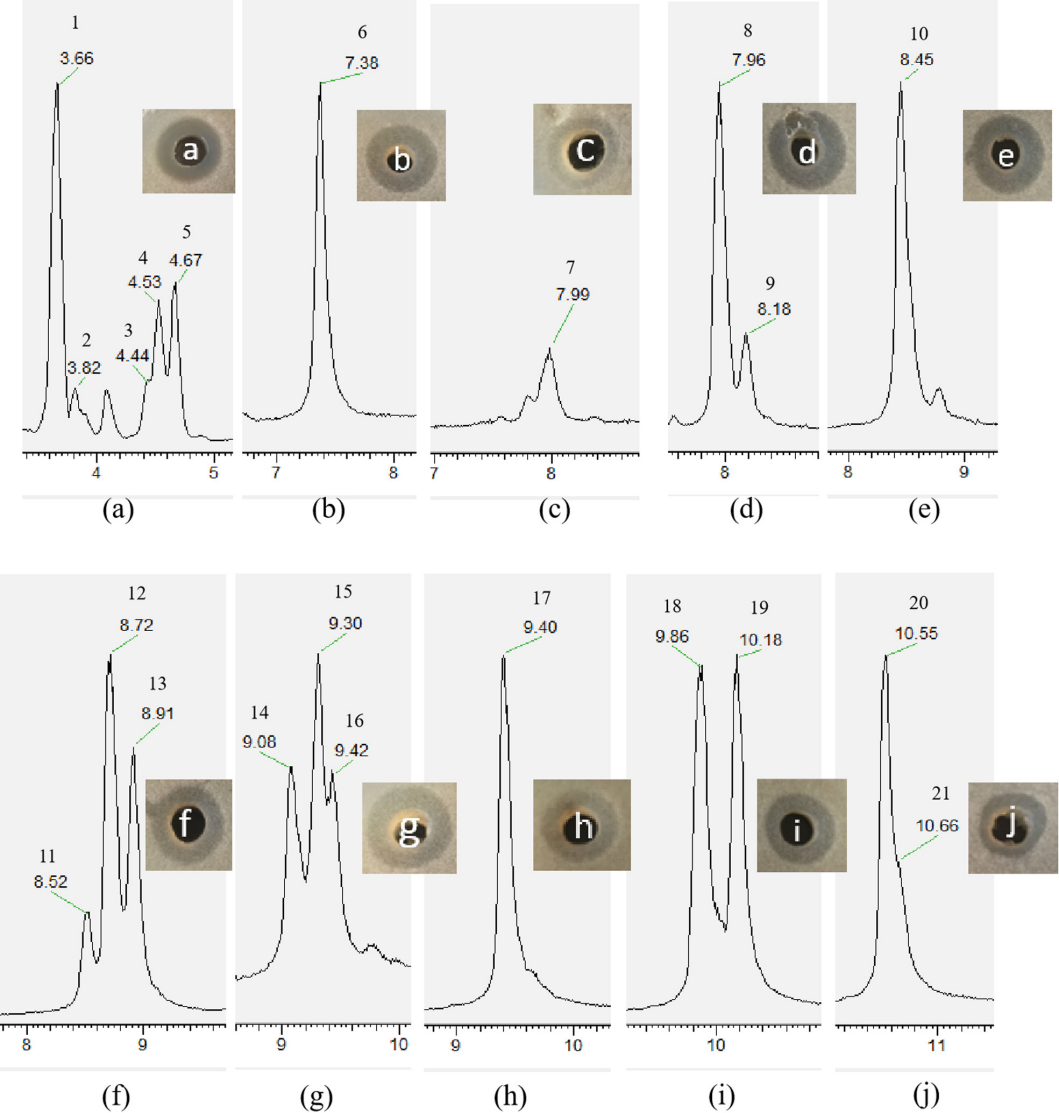
UPLC and anti-gray-mold activity analysis of the 10 fractions from *B. amyloliquefaciens* WS-8.

The antimicrobial compounds were enriched by Amberlite XAD-7HP from the cell-free supernatant and isolated by HPLC. Using gray mold as indicator bacteria, we tested all of the HPLC fractions. We found 25 fractions with anti-gray-mold activity. The Thermo Fisher UltiMate 3000 UPLC/Q-Exactive Orbitrap MS system was employed to analyze the above 25 fractions, and we found many fractions containing the same compound. Ten of these fractions (a, b, c, d, e, f, g, h, i, and j) containing non-repeating substances for further analysis were chosen, and 21 compounds showing anti-gray-mold activity were identified (Fig. 5).

Using MS to elucidate the exact molecular weight of these 21 compounds, we identified 14 lipopeptides belonging to two main types: iturin and fengycin (Table 4). Many of the components have similar charge-to-mass ratios (*m/z*), so we analyzed their secondary mass spectra one by one. All of the compounds in fraction (a) were identified as iturins. Compound 1 (*m/z* ratio of the protonated molecules [M+H]^+^ molecule is 1043.55), with the characteristic fragment ions of which have *m/z* ratios of 212.10, 392.16, 638.39, 801.44, 915.50, and 932.51 (Fig. S1), was identified as C_14_ iturin A by comparison with Xu’s data [7]. The fragment patterns of compounds **2** and **3** (Fig. S2), both with an *m/z* ratio of the protonated [M+H]^+^ molecule of 1044.53, show that their characteristic fragment ions are basically identical and they would be identified as the same molecule by mass spectrometry. However, due to the different retention time of the liquid phase, it can be concluded that these two compounds may be derivatives of C_14_ iturin B. Compounds 4 and **5**, both with an *m/z* ratio of the protonated [M+H]^+^ molecule of 1057.56, also have similar fragment patterns (Fig. S3), indicating that these two compounds may be derivatives of C_15_ iturin A.

The remaining 16 compounds were identified as fengycins. Fengycin A and fengycin B are different in their sixth amino acids of cyclic octapeptide (Ala and Val respectively). So, in the process of mass spectrometry, the daughter ion of MS peaks are different. Therefore, ion fragments (*m/z* 1080) and ion fragments (*m/z* 1108) are usually used as characteristic fragment ions to distinguish fengycin A and fengycin B. In this research, their molecular ion peaks mainly exist in the form of doubly charged [M+2H]^2+^ and singly charged [M+H]^+^ ions; most are doubly charged ions. Therefore, their fragment patterns of doubly charged ions were used in the following analysis.

Compounds 6, 17, 18, 19, 20, and 21 have unique *m/z* ratios. After analysis of their secondary ion mass spectra (Fig. S4), they were identified as C_14_ fengycin A, C_16_ fengycin B, C_17_ fengycin B, C_15:1_ fengycin A, C_16:1_ fengycin A, and C_15:1_ fengycin B, respectively. The carbon chains of compounds 19, 20, and 21 all contain a double bond.

Besides, three compounds (7, 9, and 10), with an *m/z* ratio of the protonated [M+H]^+^ molecule of 1449.78, were identified as C_15_ fengycin A. Upon analisis of secondary ion mass spectra of the doubly charged ions, we found that these three compounds had similar fragments (Fig. S5). Therefore, these three compounds may be derivatives of C_15_ fengycin A.

Compounds 8, 12, and 13 have similar molecular ion peaks (*m/z* ratio of the protonated [M+H]^+^ molecules is 1463.79), but the characteristic ion peaks in their secondary mass spectra (Fig. S6) are quite different. Compound 8, containing doubly charged fragment ions of *m/z* 554.79, equal to the characteristics fragment ions of fengycin B, was identified as C_14_ fengycin B. Compounds 12 and 13, containing doubly charged fragment ions of *m/z* 540.77, equal to the characteristics fragment ions of fengycin A, were identified as C_16_ fengycin A.

The last four compounds (11, 14, 15, and 16) show a similar molecular ion peak of the protonated [M+H]^+^ fragment at 1477.81, but their characteristic fraction ions (Fig. S7) are also different. Compound 11, containing doubly charged fragment ions of *m/z* 554.79, equal to the characteristics fragment ions of fengycin B, was identified as C_15_ fengycin B. Compounds 14, 15, and 16, containing doubly charged fragment ions of *m/z* 540.77, equal to the characteristic fragment ions of fengycin A, were identified as C_17_ fengycin A.

## Discussion

Plant disease has always been an important problem in agriculture. *B. cinerea*, an airborne plant pathogenic fungus that causes gray mold disease, affecting more than 200 crop species worldwide, is one of the most important problems in agricultural production [30]. The problem is most serious in vegetables and fruits, such as tomato, cucumber, and strawberry, causing huge economic losses [31]. The application of chemical pesticides has for a long time been the primary strategy for the prevention and control of plant diseases, but the use of chemical pesticides brings a series of problems, such as environmental pollution, so biological control has gradually become an appealing alternative. Many strains of *Bacillus* spp. are considered as safe microorganisms in agriculture [32]. They have the potential to produce more than two dozen antibiotics with an amazing variety of structures, and many of these substances have been successfully used in agricultural and industrial applications [33, 34].

*B. amyloliquefaciens* can inhibit a variety of plant pathogens and is widely used in agriculture. Since *B. amyloliquefaciens* FZB42 [5] was sequenced, more than 60 strains of *B. amyloliquefaciens* have been sequenced (including the genome reported in this study). Belbahri *et al*. analyzed the genomic information of 48 strains of *B. amyloliquefaciens*. They found that these strains have various secondary metabolite synthesis gene clusters and that obvious differences exist between strains [6]. The genome of this study was also discussed in their analysis. Through antiSMASH analysis, we found that the WS-8 genome contains seven gene clusters similar to known antibacterial biosynthetic gene clusters, which indicates that WS-8 bacteria may produce these antibacterial agents or their homologues. By using the FPKM values of the transcriptome, we found that core genes of six gene clusters, which are homologous to bacillibactin, fengycin, bacillaene, difficidin, macrolactin, and surfactin biosynthetic gene clusters, were all expressed. This indicates that the secondary metabolites of *B. amyloliquefaciens* WS-8 may include many bioactive substances.

Ten fractions (a, b, c, d, e, f, g, h, i, j, and k) containing 21 compounds with strong anti-gray-mold activity were fractionated from the cell-free supernatants of *B. amyloliquefaciens* WS-8 culture by a combination of Amberlite XAD-7HP resin and reversed phase chromatography. Compared with published LC-MS/MS data, we found that these active compounds are identified as iturins and fengycins. The iturin compounds identified in this study were C_14_ iturin A, C_14_ iturin B and C_15_ iturin A; two derivatives of C_14_ iturin B and C_15_ iturin A were found. Iturin compounds have been found in many strains of *B. amyloliquefaciens* and their applications in biological control have also been reported [32, 35]. In this study, 11 fengycin compounds were identified, *i.e.*, C_14_ fengycin A, C_15_ fengycin A, C_16_ fengycin A, C_17_ fengycin A, C_15:1_ fengycin A, C_16:1_ fengycin A, C_14_ fengycin B, C_15_ fengycin B, C_16_ fengycin B, C_17_ fengycin B, and C_15:1_ fengycin B. Among these, derivatives of C_15_ fengycin A, C_16_ fengycin A, and C_17_ fengycin A were found. In addition, three fengycins with a double bond were found, *i.e.*, C_15:1_ fengycin A, C_16:1_ fengycin A, and C_15:1_ fengycin B. Xu *et al*. analyzed the metabolites of B. siamensis JFL15, and they reported that fengycins were the main active substances [7]. The above results suggest that the strain WS-8 produces many bioactive derivatives.

Surfactin does not have antifungal activity, but it can enhance the antifungal activity of other lipopeptide, especially iturin [36, 37]. In this research, although core genes of surfactin biosynthesis cluster were under high expression level, but no surfatin was detected. Because of no anti-graymold activity, the fractions containing surfactin probably were abandoned after the active test.

In conclusion, the genome of *B. amyloliquefaciens* WS-8 was sequenced and annotated; based on the genome data, the active substances of this strain were isolated and identified. A total of 21 known metabolites were identified, five of which were iturins and 16 were fengycins, according to MS/MS spectra data. Our results indicate that *B. amyloliquefaciens* WS-8 can be used as an effective biocontrol agent in agriculture. Future studies will be necessary to further clarify the effects of this strain on pathogenic microorganisms in different crops.

## Supplemental Materials



Supplementary data for this paper are available on-line only at http://jmb.or.kr.

## Figures and Tables

**Table 1:** General genome features of *B. amyloliquefaciens* WS-8.

Feature	Value
Genome size (bp)	3929787
GC content [%]	45.6
Predicted genes	3895
Protein coding genes (CDSs)	3777
Pseudo genes	107
tRNA genes	86
rRNA genes	27
ncRNAs genes	5

**Table 2:** Secondary metabolite clusters in *B. amyloliquefaciens* WS-8 identified by antiSMASH 3.0.

Cluster	Type	From	To	Most similar known biosynthetic gene cluster (percent of similarity)	MIBiG BGC-ID
1	Polyketide	106469	206922	Difficidin (93% )	BGC0000176_c1
2	Polyketide	321899	363008	NA	NA
3	Terpene	426326	448209	NA	NA
4	Nonribosomal peptide	473436	611237	Fengycin (93%)	BGC0001095_c1
5	Nonribosomal peptide	675868	778542	Bacillaene (92%)	BGC0001089_c1
6	Polyketide	1004652	1090557	Macrolactin (90%)	BGC0000181_c1
7	Lantipeptide	1259447	1288335	NA	NA
8	Terpene	1408827	1429567	NA	NA
9	Polyketide	1511611	1552855	Butirosin (7%)	BGC0000693_c1
10	Microcin	1609461	1629609	NA	NA
11	Microcin	1878888	1899036	NA	NA
12	Nonribosomal peptide	2089305	2154712	Surfactin (78%)	BGC0000433_c1
13	Microcin	2298675	2324765	NA	NA
14	Microcin	2369207	2395180	NA	NA
15	Microcin	2435066	2455214	NA	NA
16	Microcin	2456802	2476950	NA	NA
17	Other	2776305	2817723	Bacilysin (85%)	BGC0001184_c1
18	Nonribosomal peptide	3354032	3405823	Bacillibactin (92%)	BGC0000309_c1
19	Microcin	3470058	3490206	NA	NA

**Table 3:** Transcriptomic features of *B. amyloliquefaciens* WS-8.

FPKM Interval	Gene counts	Percentage
0~1	277	60.2%
1~10	0	0.00%
10~500	3,045	80.83%
>500	495	13.14%

The transcripts were assessed based on FPKM values: high expression (FPKM ≥ 500), medium expression (10 ≤ FPKM< 500), low expression (1 ≤ FPKM < 10), and no expression (FPKM < 1).

**Table 4:** The *m/z* value of active substance detected by Q-Exactive Orbitrap MS.

Fraction no.	Compound no.	*m/z*	*m/z*	Characteristic fragment ions	Retention time (min)	Identification
		[M+H]^+^	[M+Na]^+^			
a	**1**	1043.5474	1065.5284	-	3.66	C_14_Iturin A
	**2**	1044.5328	1066.5145	-	3.82	C_14_ Iturin B
	**3**	1044.5334	1066.5149	-	4.44	C_14_ Iturin B
	**4**	1057.5643	1079.5454	-	4.53	C_15_Iturin A
	**5**	1057.5636	1079.5449	-	4.67	C_15_Iturin A
b	**6**	1435.766	1457.7474	540.77	7.38	C_14_ Fengycin A
c	**7**	1449.7848	1471.7635	540.77	7.99	C_15_ Fengycin A
d	**8**	1463.7975	1485.7784	554.79	7.96	C_14_Fengycin B
	**9**	1449.7836	1471.7632	540.77	8.18	C_15_ Fengycin A
e	**10**	1449.7816	1471.7626	540.77	8.45	C_15_ Fengycin A
f	**11**	1477.8138	1499.7944	554.79	8.52	C_15_ Fengycin B
	**12**	1463.7969	1485.7777	540.77	8.72	C_16_ Fengycin A
	**13**	1463.7974	1485.7789	540.77	8.91	C_16_ Fengycin A
g	**14**	1477.8138	1499.7888	540.77	9.08	C_17_ Fengycin A
	**15**	1477.814	1499.7932	540.77	9.3	C_17_ Fengycin A
	**16**	1477.8157	1499.7963	540.77	9.42	C_17_ Fengycin A
h	**17**	1491.8285	1513.8091	554.79	9.4	C_16_ Fengycin B
i	**18**	1505.8439	1527.825	554.79	9.86	C_17_ Fengycin B
	**19**	1447.8036	1469.7852	540.77	10.18	C_15:1_ Fengycin A
j	**20**	1461.8197	1483.8	540.77	10.55	C_16:1_ Fengycin A
	**21**	1475.8328	1497.8135	554.79	10.66	C_15:1_ Fengycin B
