# Supplementary material for: Genomics and LC-MS Reveal Diverse Active Secondary Metabolites in Bacillus amyloliquefaciens WS-8
Source: J Microbiol Biotechnol. 2019 Oct 10;30(3):417–26. doi: 10.4014/jmb.1906.06055 (PMC9728402; doi:10.4014/jmb.1906.06055)
Supplement: Supplementary file 1 [file JMB-30-3-417-supple.pdf]

J. Microbiol. Biotechnol.
https://doi.org/10.4014/jmb.1906.06055

jmb

1

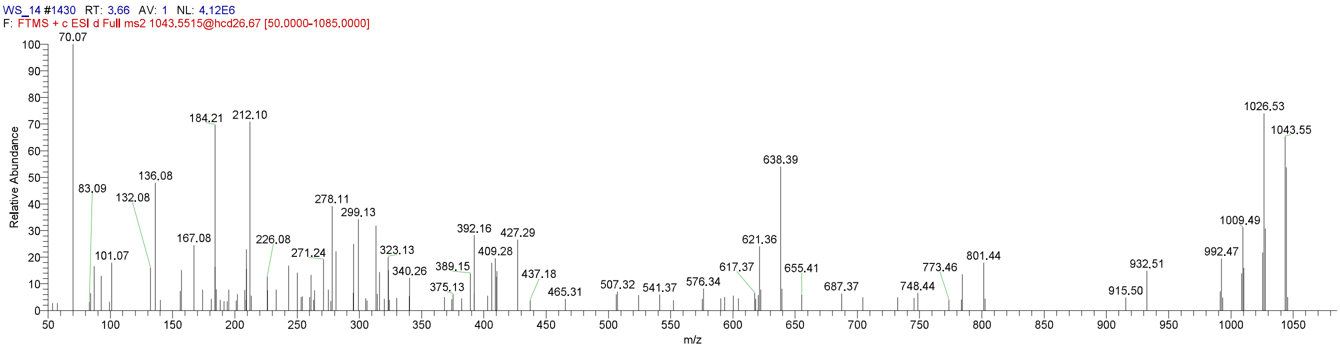

Figure S1 MS/MS spectra of compounds 1

2

3

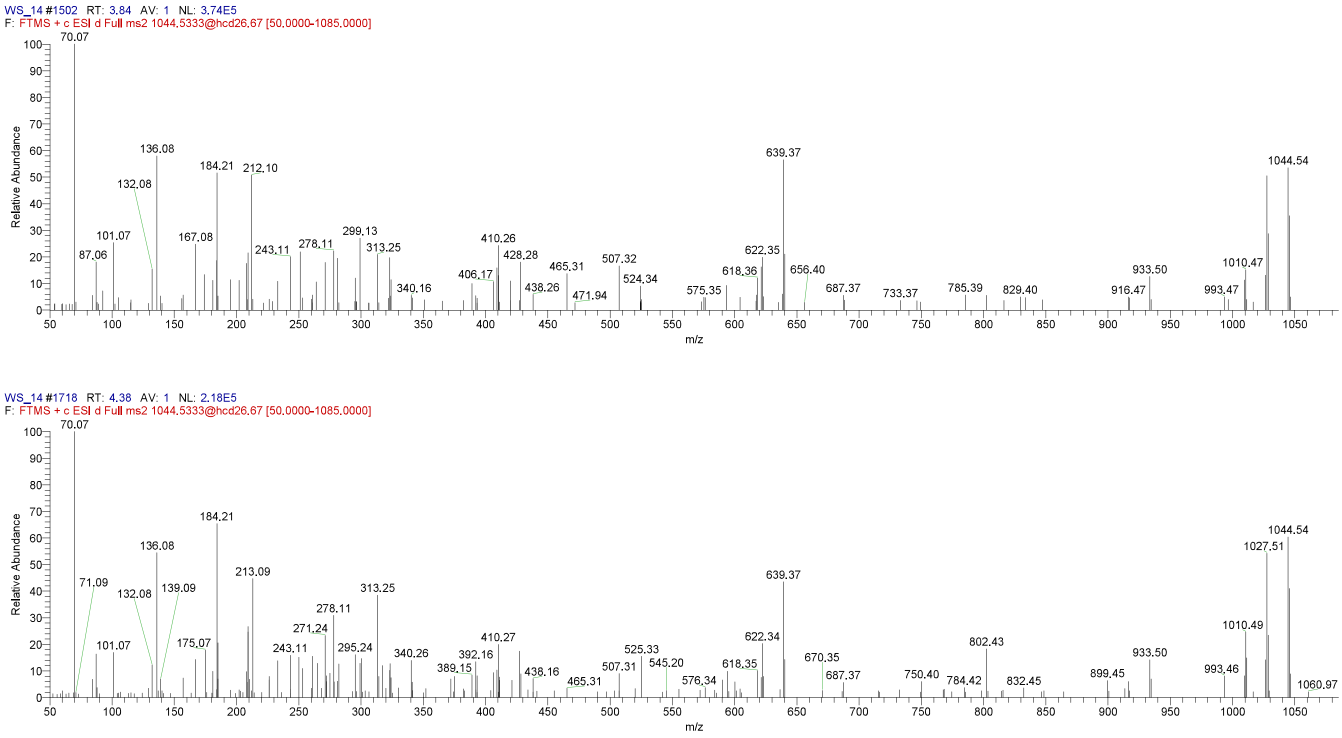

Figure S2 MS/MS spectra of compounds 2 and 3

4

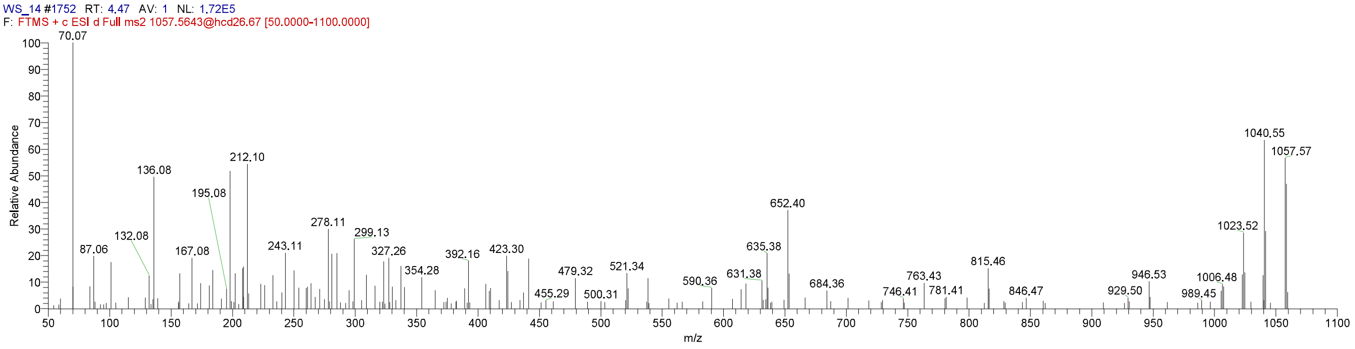

5

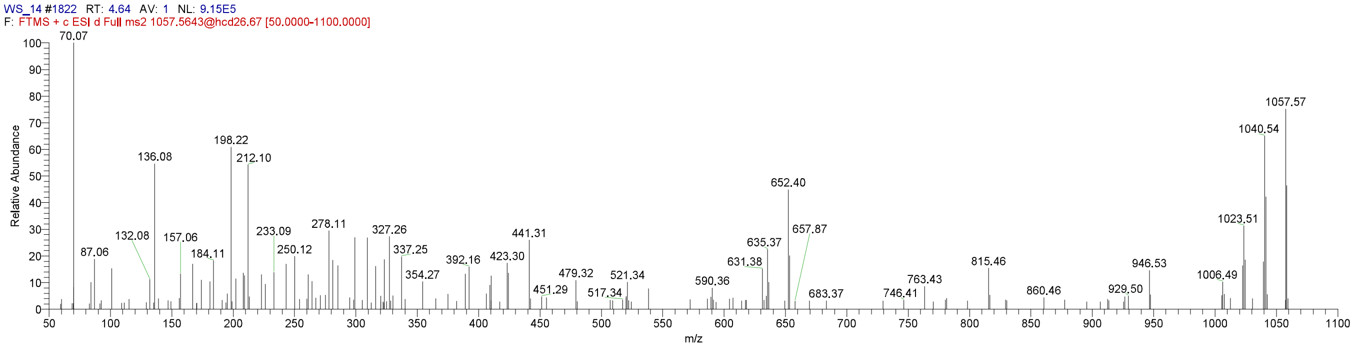

Figure S3 MS/MS spectra of compounds 4 and 5

6

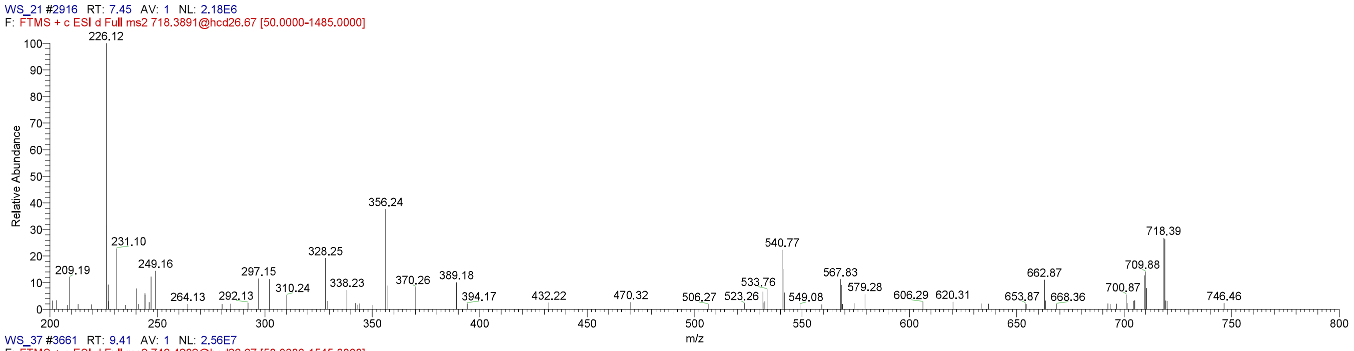

17

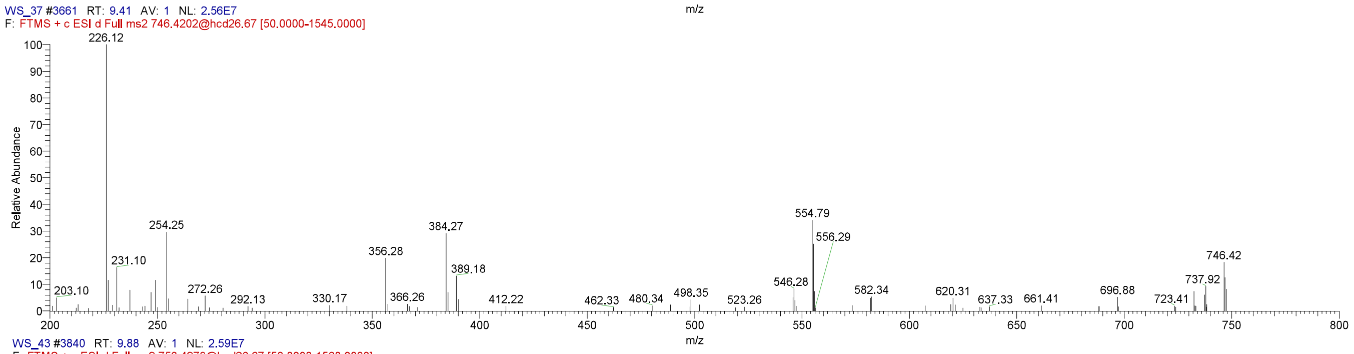

18

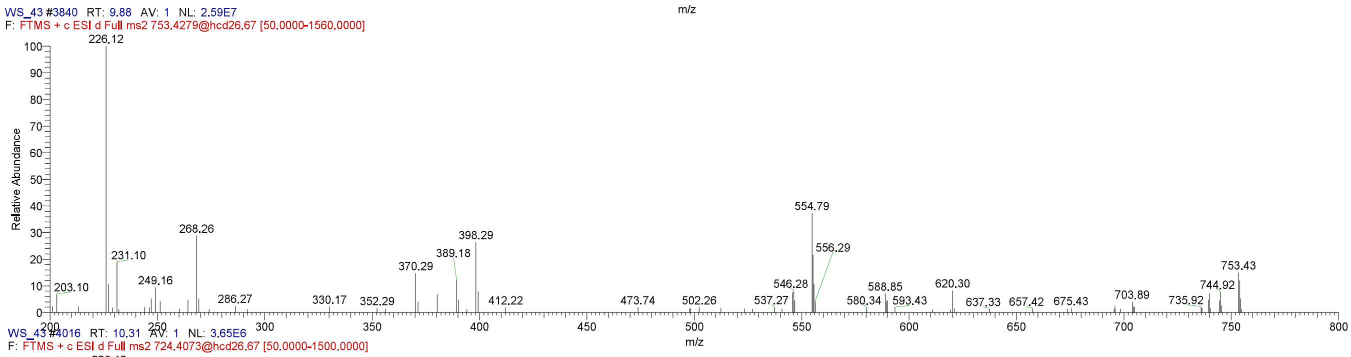

19

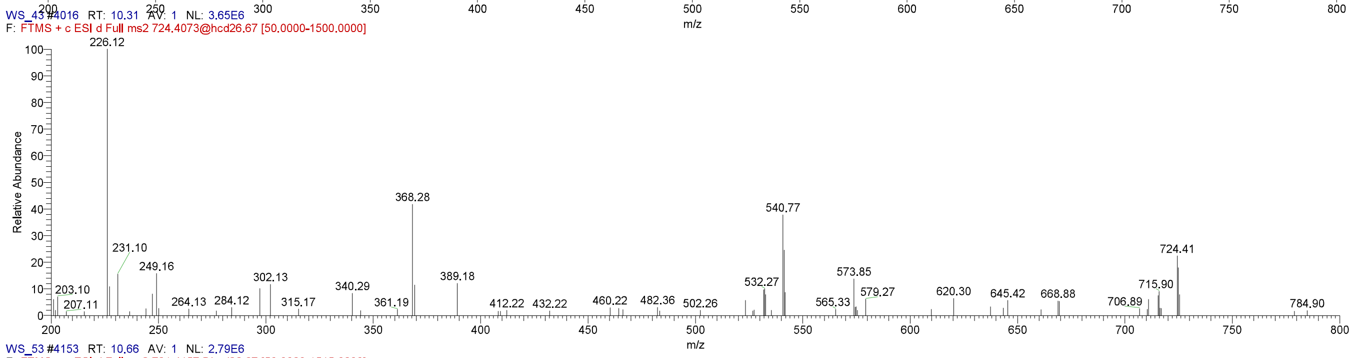

20

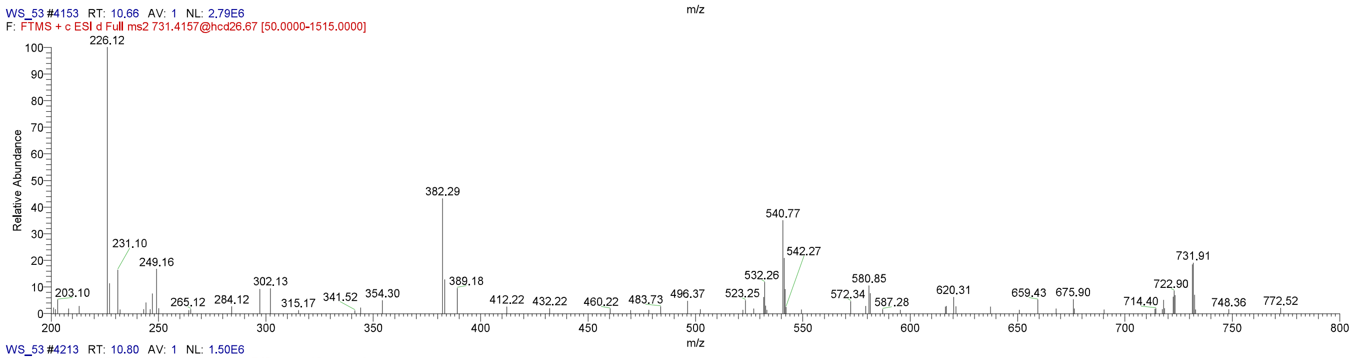

21

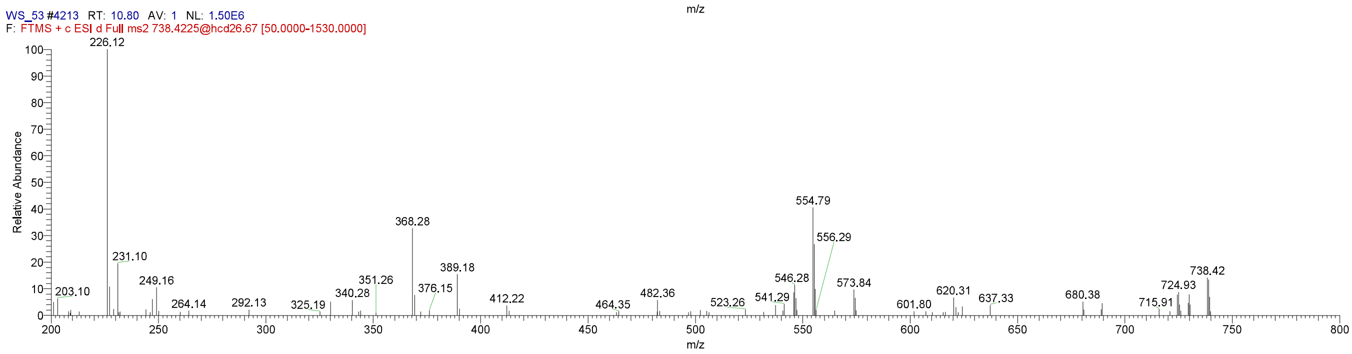

Figure S4 MS/MS spectra of compounds 6, 17, 18, 19, 20 and 21

7

9

10

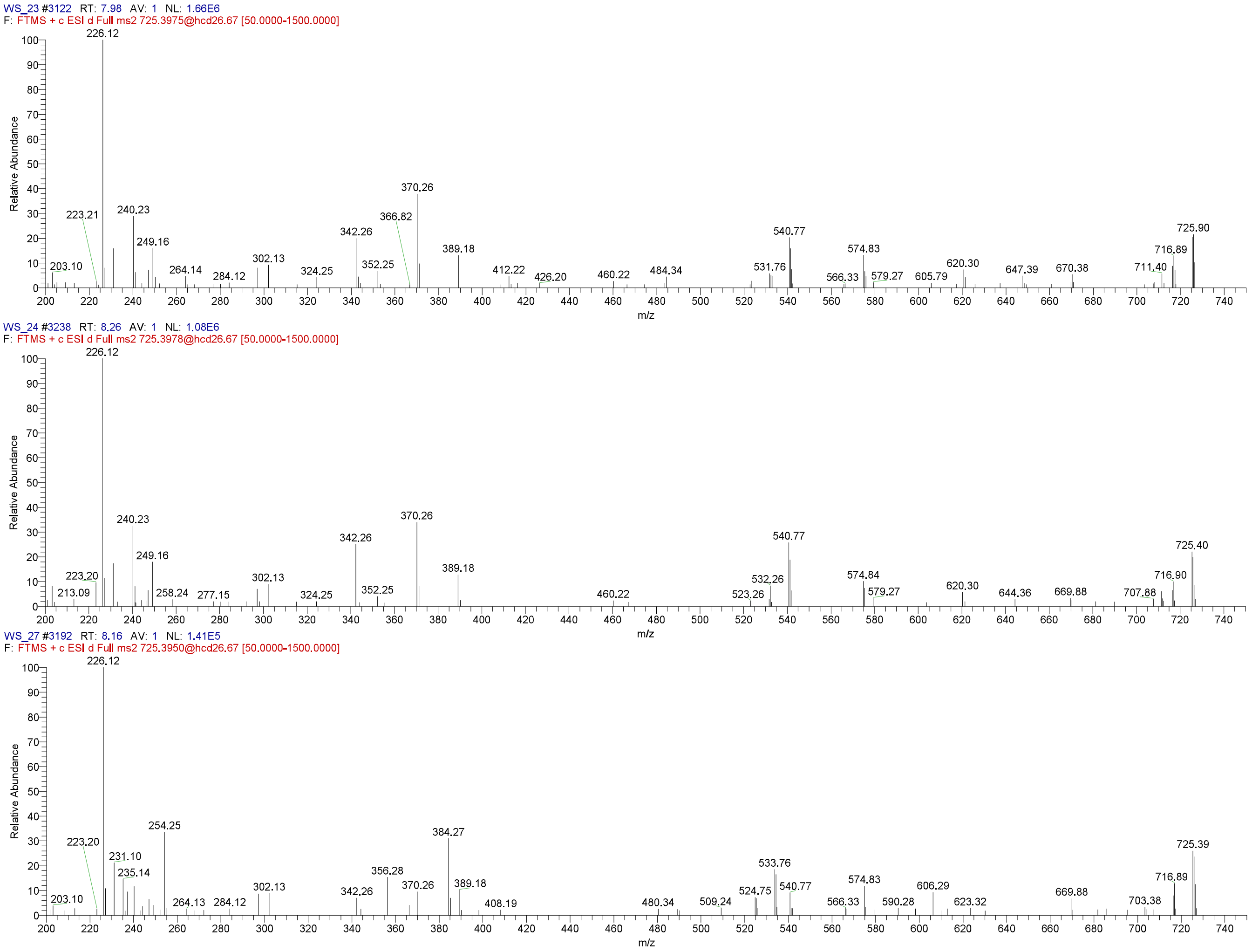

Figure S5 MS/MS spectra of compounds 7, 9 and 10

8

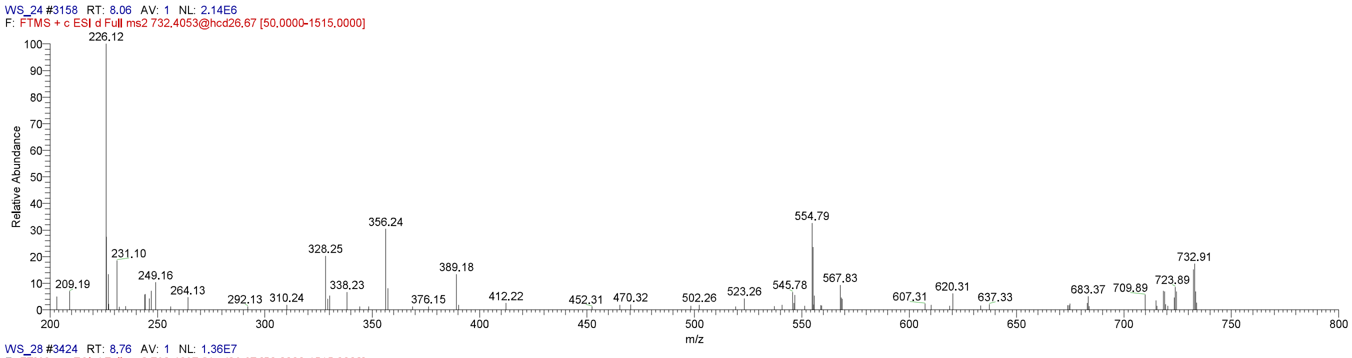

12

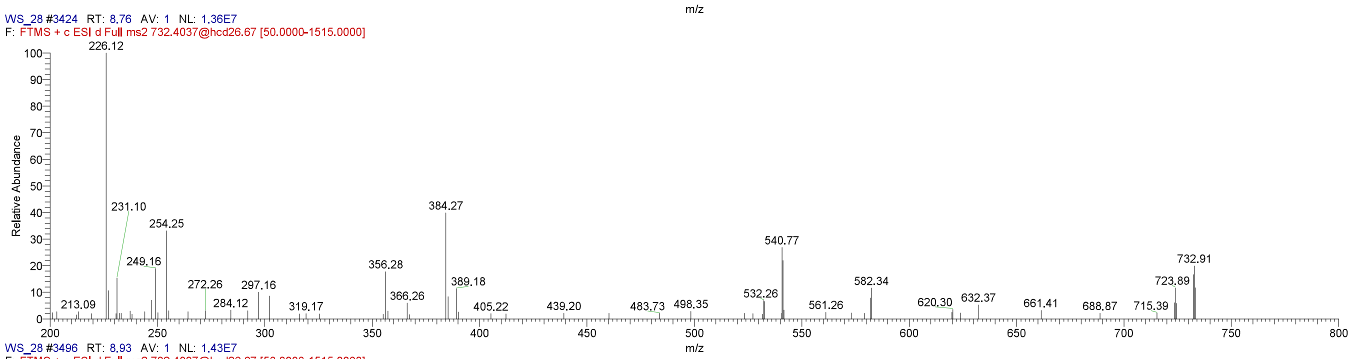

13

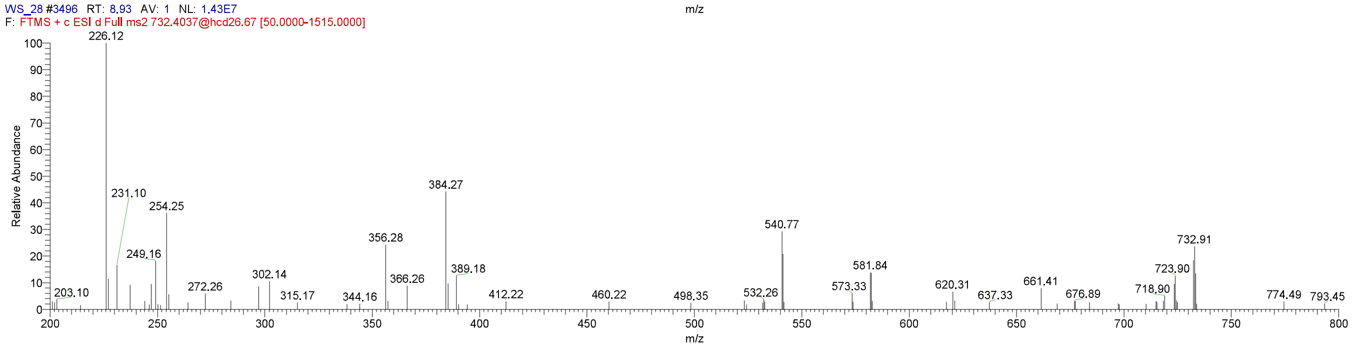

Figure S6 MS/MS spectra of compounds 8, 12 and 13

11

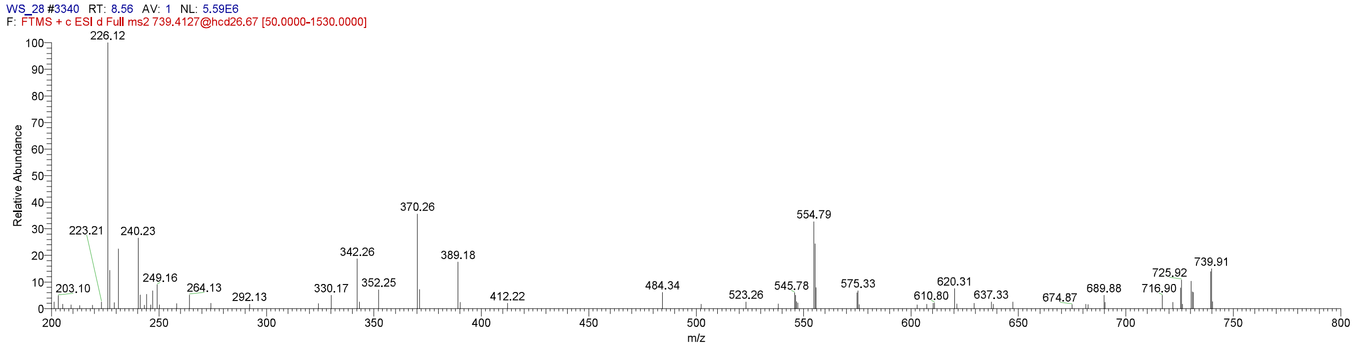

14

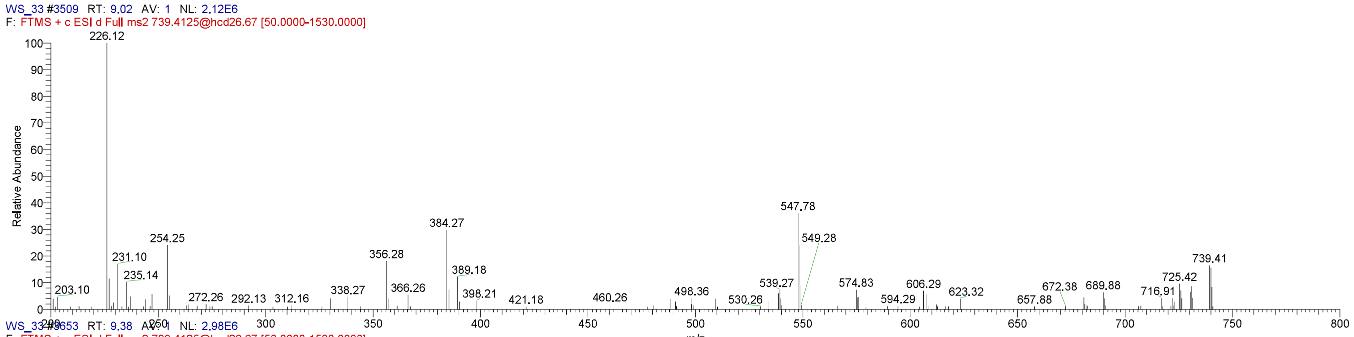

15

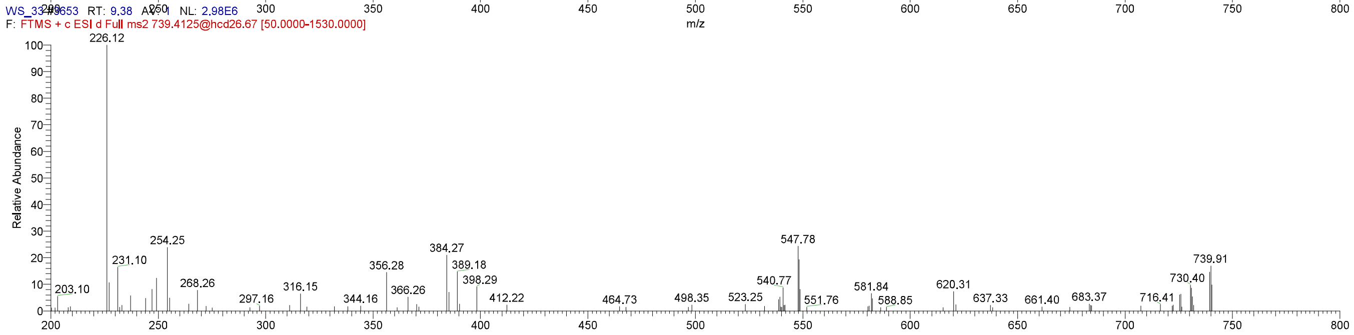

16

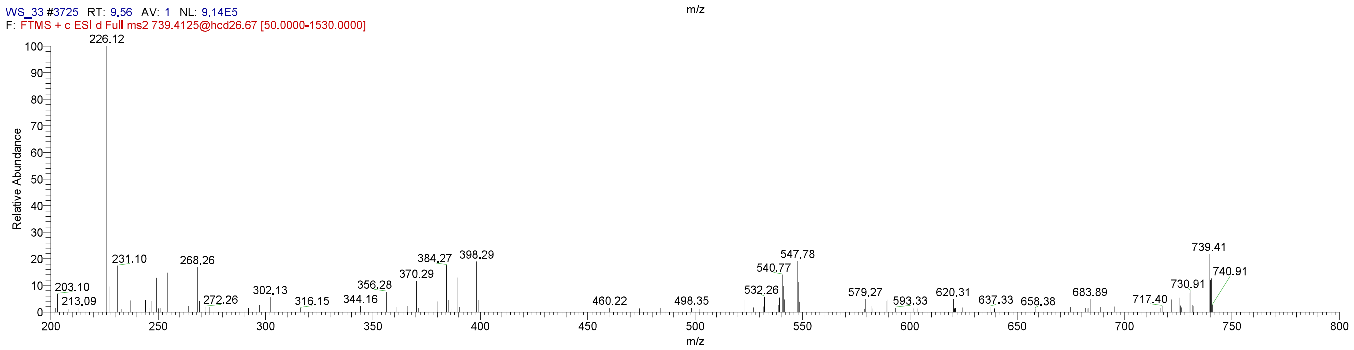

Figure S7 MS/MS spectra of compounds 11, 14, 15 and 16
